# *Helicobacter pylori*-related precancerous lesions in Turkey: a retrospective endoscopic surveillance study

**DOI:** 10.3325/cmj.2020.61.319

**Published:** 2020-08

**Authors:** Bilge Bas, Bulent Dinc

**Affiliations:** 1 Department of Gastroenterology, Health Sciences University, Antalya Training and Research Hospital, Antalya, Turkey; 2 Department of General Surgery, Health Sciences University, Antalya Training and Research Hospital, Antalya, Turkey

## Abstract

**Aim:**

To assess the relationship between *Helicobacter pylori* (*H. pylori*) infection and atrophic gastritis (AG) and intestinal metaplasia (IM) development and to assess the rate of dysplasia or gastric cancer development in patients with AG and/or IM.

**Methods:**

This retrospective endoscopic follow-up study enrolled 2214 patients. The patients were followed for at least five years between 2007 and 2017 at the Department of Endoscopy at Antalya Ataturk Government Hospital. The results of third-year and five-year surveillance biopsy were assessed.

**Results:**

The mean follow-up time was 7.77 ± 2.78 years. *H. pylori* was histologically assessed in 1417 (64.6%) patients. Of 198 patients with severe *H. pylori* infection, 32 (16%) and 139 (70.3%) developed extensive AG and extensive IM, respectively. There was a significant relationship between *H. pylori* density and AG and IM degrees. High grade dysplasia, early gastric cancer, and advanced gastric cancer were diagnosed in 73 patients with median age 58.2 (28-80) years, and the incidence rate was 3.29% (73/2214). The annual incidence of gastric neoplastic lesions was 0.46% in total, 0.08% for early GC, and 0.02% for advanced gastric cancer.

**Conclusions:**

*H. pylori* infection has an important role in the development of AG and IM. *H. pylori* density is directly related to atrophy and metaplasia degree.

*Helicobacter pylori* (*H. pylori*) is a widely spread bacterium causing atrophic gastritis (AG), intestinal metaplasia (IM), dysplasia, and eventually gastric cancer (1). Gastric cancer ranks fourth in terms of prevalence and second in terms of the number of cancer-related deaths. The most common gastric cancer type is intestinal type adenocarcinoma, which is closely associated with *H. pylori* infection (2).

In *H. pylori*-related AG, the glandular tissue in the stomach disappears, while in IM the surface foveolar and glandular epithelium of the gastric mucosa is replaced with intestinal epithelium. In general, metaplasia is the cell’s protective response to changing ambient conditions, since sulfomucins and sialomucins in the intestinal mucosa are resistant to bacterial enzymes. AG and IM are considered the precursors of gastric cancer (3-5). It is widely believed that they could be ameliorated by *H. pylori* eradication (6). *H. pylori* infection is a risk factor for developing gastric carcinoma (1). However, only a few infected patients develop gastric cancer, which is why it is important to identify the *H. pylori* gastritis characteristics that increase the risk for gastric carcinoma. We investigated whether a simple comparison of the gastritis grade and chronic inflammation in the antrum and corpus might help identify patients with *H. pylori* gastritis with an increased cancer risk. To answer this question, we retrospectively assessed whether patients with AG and/or IM with *H. pylori* developed gastric cancer or dysplasia in at least 5-year follow up.

## PATIENTS AND METHODS

In this endoscopic follow-up study, we retrospectively reviewed the charts of patients who underwent first gastroscopy at the Department of Endoscopy at Antalya Ataturk Government Hospital. The gastroscopy was performed due to epigastric pain, abdominal bulge, nausea, vomiting, and heartburn with at least one premalignant lesion in the stomach biopsy. The patients were followed up for more than 5 years between 2007 and 2017. The exclusion criteria included stomach adenocarcinoma, mucosal associated lymphoid tumor, gastrectomy, previous *H. pylori* eradication therapy, and age under 18 years. Baseline and surveillance endoscopies were performed by experienced gastroenterologists with Fujinon EG 450 WL5 (Fujifilm Europe GmbH, Dusseldorf, Germany) and Pentax EG-2980 K unit (Pentax Medical, Montvale, NJ, USA).

All patients underwent endoscopic examination of the esophagus, stomach, and duodenum. During the first endoscopy, two non-targeted antral and two gastric body biopsies were performed by using Olympus biopsy forceps (Olympus Europa, Hamburg, Germany). All biopsy specimens were fixed in 10% formalin and examined at the Pathology Department. *H. pylori* density was determined with Giemsa dye.

Histopathological classification was made according to the updated Sydney System (7) (Table 1). All biopsy samples were evaluated for *H. pylori*, IM, AG, and dysplasia and graded according to their density as absent, mild (grade 1), moderate (grade 2), and severe (grade 3). The dysplasia degree was assessed using the Vienna system; dysplasia was graded as low-grade to high-grade or neoplasia. A control endoscopy was performed after 3 years, with biopsies performed and evaluated in the same manner as at baseline. *H. pylori* infection was treated by triple (proton pump inhibitor, clarithromycin, and amoxicillin) and quadruple therapy (proton pump inhibitor, bismuth, metronidazole, and tetracyline). The patients underwent endoscopic examination after having given written and verbal consent. This study was approved by the institutional review board of the University of Health Sciences Antalya Training and Research Hospital (13.08.2020-12/10).

**Table 1:** Updated Sydney System (8)

Histologic properties	Definition	Mild	Moderate	Severe
Chronic inflammation	Lymphocyte and plasma cell propria	+	++	+++
Neutrophil activation	Neutrophil infiltration in the lamia propria or superficial epithelium	<l/3	1/3-2/3	>2/3
Glandular atrophy	Loss of corpus and antral glands	+	++	+++
Intestinal metaplasia	Intestinal metaplasia in the mucosal epithelium	<l/3	1/3-2/3	>2/3
*Helicobacter pylori*	*Helicobacter pylori* intensity in the epithelium	+	++	+++

### Statistical analysis

The Pearson and Kruskal-Wallis tests were used to evaluate the differences in outcome proportions (at least LGD or at least HGD) and to assess the differences in patients’ age, sex, Hp infection prevalence, and disease duration. Survival analysis was performed to define the rate of progression to outcome at the third- and fifth-year follow up endoscopy. Outcome was considered as progression to lesions as severe as LGD. Age was presented with median, minimum, and maximum value. The level of significance was set at 0.05. The analysis was conducted with SPSS, version 17.0 (SPSS, Chicago, IL, USA).

## RESULTS

The study enrolled 2214 patients (1285 or 58.3% male). Men and women did not significantly differ in median age (54.5 [18-64] women and 58.5 [18-69] men, *P* = 0.495) (Table 2). The mean follow-up period was 7.77 ± 2.78 years. *H. pylori* was histologically confirmed in 1417 (64.6%) of 2214 patients.

**Table 2:** Age and sex of participants with and without *Helicobacter pylori* infection

n (%)	*H. pylori*-negative (n = 797)	*H. pylori*-positive (n = 1417)	total (n = 2214)
**Age (years)**			
<30	133 (55.2)	108 (44.8)	241
30-49	261 (36.7)	451 (63.3)	712
50-59	146 (26.5)	405 (73.5)	551
>60	257 (36.2)	453 (63.8)	710
**Sex**			
female	393 (42.4)	536 (57.6)	929 (41.7)
male	404 (31.5)	881 (68.5)	1285 (58.3)

*H. pylori* density was significantly associated with chronic inflammation degree (*P* = 0.001), atrophy degree (*P* = 0.0001), and IM degree (*P* = 0.0028). Significantly more patients with *H. pylori* had chronic inflammation, atrophy, and IM compared with patients without *H. pylori* (Table 3, Table 4, Table 5). A total of 493 patients with *H. pylori* infection simultaneously had histologic features of IM and AG.

**Table 3:** The prevalence of chronic inflammation in patients with and without *Helicobacter pylori* infection

	No. (%) of patients				
	***H. pylori*-negative** **(N = 797)**	***H. pylori*-positive** **(N = 1417)**			
	**none**	**mild (+)**	**moderate (++)**	**severe (+++)**	**total**
**Chronic inflammation**					
mild	605 (75.9)	654 (81.3)	226 (55)	8 (4)	888 (62.6)
moderate	151 (18.9)	121 (15.5)	140 (33.9)	64 (32.3)	325 (22.9)
severe	41 (5.2)	32 (3.2)	46 (11.1)	126 (68.7)	204 (14.3)
total	797	807 (56.9)	412 (29)	198 (13.9)	1417
Odds ratio	3.8				
95% confidence interval	3.47-4.2				
*P*	<0.01				

**Table 4:** The prevalence of gastric atrophy in patients with and without *Helicobacter pylori* infection

	No. (%) of patients				
	*H. pylori*-negative (N = 797)	*H. pylori*-positive (N = 1417)			
	none	mild (+)	moderate (++)	severe (+++)	total
**Gastric atrophy**					
none	713 (89.4)	582 (72)	256 (62)	86 (43)	924 (65.2)
mild	84 (10.5)	225 (28)	0	0	225 (15.8)
moderate	0	0	113 (27.6)	80 (41)	193 (13.6)
severe	0	0	43 (10.4)	32 (16)	75 (5.29)
total	797	807 (56.9)	412 (29)	198 (13.9)	1417
Odds ratio	3.3				
95% confidence interval	3.13-3.47				
*P*	<0.01				

**Table 5:** The prevalence of intestinal metaplasia in patients with and without *Helicobacter pylori* infection

	No. (%) of patients				
	*H. pylori*-negative (N = 797)	*H. pylori*-positive (N = 1417)			
	none	mild (+)	moderate (++)	severe (+++)	total
Intestinal metaplasia					
none	713 (89.4)	583 (72)	0	0	583 (41.1)
mild	84 (10.5)	170 (21.4)	246 (59.7)	0	416 (29.3)
moderate	0	54 (6.6)	166 (40.3)	139 (70.3)	359 (25.3)
severe	0	0	0	59 (29.7)	59 (4.1)
total	797	807 (56.9)	412 (29)	198 (13.9)	1417
Odds ratio	5.6				
95% confidence interval	5.12-5.96				
*P*	0.0028				

IM was most frequently detected by follow-up endoscopy. High grade dysplasia (HGD), early gastric adenocarcinoma (early gastric cancer), and advanced gastric adenocarcinoma (advanced gastric cancer) were diagnosed in 73 patients (24 women, median age 58.2 [49-68] years), with the crude incidence rate of 3.29% (Table 6). These patients were followed-up more closely. In 28 of the 73 gastric cancer patients, biopsy was taken from the suspicious areas of IM or visible lesions. Biopsies from visible lesions were evaluated as high-grade advanced GC in 3 patients, as IM in 14 patients, as early GC in 4 patients, and as high-grade dysplasia in 6 patients.

**Table 6:** Histological lesions found at outcome endoscopic biopsy at the first, third, and fifth year after diagnosis according the lesion found at entry biopsy

n (%)	First biopsy	Third-year biopsy				Fifth-year biopsy			
		LGD	HGD	EGC	AGC	LGD	HGD	EGC	AGC
**Atrophy**									
+	225	24 (10.6)	1 (0.4)	0	0	29 (12.8)	4 (1.7)	0	0
++	193	21(10.8)	3 (1.5)	0	0	32 (16.5)	12 (6.2)	0	0
+++	75	8(10.6)	2 (2.6)	0	0	11 (14.6)	6 (8)	0	2 (2.6)
**Metaplasia**									
+	416	21 (5)	4 (0.9)	0	0	32 (7.6)	14 (3.3)	0	0
++	359	18 (5)	5 (1.3)	0	0	28 (7.7)	19 (5.2)	0	5 (1.3)
+++	59	4 (6.7)	2 (3.3)	0	0	7 (11.8)	9 (15.2)	0	2 (3.3)

*LGD – low grade dysplasia; HGD – high grade dysplasia; EGC – early gastric carcinoma; AGC – advanced gastric carcinoma.

Among 73 patients with neoplastic lesions, 57 patients had lesions localized in the antrum (35 low-grade dysplasia, 14 high-grade dysplasia, 5 early GC, and 3 advanced GC). Sixteen patients had lesions localized in the gastric body (9 LGD, 4 HGD, and 3 early GC) (Table 7).

**Table 7:** Characteristics of patients who developed gastric neoplastic lesions at follow-up

	LGD	HGD	Early GC	Advanced GC
**Antrum**	35	14	5	3
**Corpus**	9	4	3	-
**Age**	49 to 68 years, median age 58.2 ± 5.74 years			
**Gender**	24 women, 49 men (*P* < 0.05)			
**Crude incidence rate**	3.29% (73/2214)			
**Average length of follow-up**	7.77 ± 2.78 year			

*LGD – low grade dysplasia; HGD – high grade dysplasia; GC – gastric carcinoma.

The annual incidence rate of total gastric neoplastic lesions per person was 0.46%. The annual incidence of LGD, HGD, early GC, and advanced GC was 0.37%, 0.18%, 0.08%, and 0.02%, respectively.

A total of 364 patients had moderate and severe IM and/or moderate and severe AG. Severe metaplasia and atrophy at first biopsy progressed at a higher rate than others, both at 3 and 5 years after diagnosis.

## DISCUSSION

In this study, at baseline biopsy, all lesions (AG, IM, LGD) were equally prevalent. The patients with AG or IM at first biopsy did not develop cancer during the following three years, and less than 5% progressed to HGD. Although we did not confirm the direct effect of *H. pylori* infection on lesion progression, it is a potential risk factor for cancer, because it causes chronic gastritis and leads to atrophy. We found no significant differences in age, sex, *H. pylori* infection prevalence, and follow up duration according to the histological diagnosis of the first lesion.

Several studies have shown that the presence and severity of *H. pylori* infection correlates with chronic gastritis, AG, and IM (8). As is well known, AG and IM are important risk factors for gastric cancer, together with genetic and other environmental factors (9). In AG cases, the risk of gastric cancer development increases with disease duration, atrophy severity, IM presence, dysplasia, epigenetic changes, and *H. pylori* infection. In 10% of cases with AG, gastric cancer can develop within 10-20 years (10-12).

A 20-year prospective study by Lahner et al showed a person-year incidence of gastric cancer in patients with AG to be 0.25% (4). Another study (13) observed 8.4% cases of gastric cancer among patients with IM and AG who were followed up for 10 years, with endoscopy being performed once a year. The risk of developing malignancy in these patients was 11% (13).

A Dutch nationwide cohort study found the annual gastric cancer incidence to be 0.1%, 0.25%, 0.6%, and 6% in 5 years in patients with AG, IM, mild, and moderate and severe dysplasia (14). East Asian countries have higher gastric adenocarcinoma prevalence than Western countries (15), and the prevalence of *H. pylori* in Turkey is similar to Asian countries. Since patients with extensive IM have a high risk of gastric cancer, they are recommended to undergo follow-up endoscopy (16-18). Similar to other studies, our study found extensive AG and/or IM to be risk factors for gastric cancer and dysplasia. As we have already noted, *H. pylori* infection is an important etiological factor for gastric cancer, and its eradication reduces the risk of cancer development (19). Some authors have also reported that IM areas in the stomach after *H. pylori* treatment convert into normal mucosa, meaning that the progression to gastric cancer can be prevented. However, complete prevention can only be achieved by *H. pylori* eradication therapy before AG and IM develop (20). Although chronic AG may be cured after bacterial eradication, AG and IM or dysplasia still present a high risk for progression to gastric cancer (21).

In the study by Kwak et al (22), most of 1833 gastric cancer patients had *H. pylori* infection. In our study, all of 73 new patients with gastric cancer had intestinal adenocarcinoma and 55 of them had *H. pylori* with IM. This result shows a significant relationship between *H. pylori* and gastric intestinal adenocarcinoma. *In H. pylori* carriers with all precancerous lesions, *H. pylori* eradication significantly reduced the development of gastric cancer (5). Our study also showed that more than half of patients with precancerous lesions were *H. pylori* positive.

These data show that *H. pylori* infection plays an important role in the development of atrophy and IM. We also observed that activity and atrophy increased with an increase in *H. pylori* density. Performing routine biopsies can contribute to *H. pylori* eradication and IM and AG treatment. However, it is very important to determine the most suitable screening intervals as early diagnosis can reduce the number of cancer deaths. European guidelines (23) recommend a screening interval of 3 years in patients with extensive AG and IM. A Korean study determined that the mean screening interval of 2.37 years was appropriate for gastric cancer detection (24). In a Japanese case-control study, patients who underwent 3-year endoscopic screening intervals had reduced gastric cancer-related mortality by 30% compared with non-screened patients (25). These studies show 2- or 3-year intervals are not superior to each other (26). Consistent with all these studies, we showed that endoscopic screening period of 3 years would be sufficient for early detection of GC for patients with extensive AG and IM.

The study is limited by retrospective and single-center design. In addition, histological diagnosis of *H. pylori* was made only by Giemsa staining, and biopsy specimens were evaluated by a single pathologist. We also obtained only two biopsies from the gastric antrum and body, which is fewer than in other studies and may have prevented us from observing some small premalignant lesions. Due to the retrospective design, we were not able to assess patients’ smoking status, consumption of salted food and alcohol, and family history, which are all known risk factors for the development of GC. The use of white light endoscopy can also be considered a limitation as new guidelines recommend the use of narrow band imaging for early detection of premalignant gastric lesions (23). Target sampling in uneven areas with narrow band imaging (NBI) increases the likelihood of finding a pathological lesion and the reliability of the study. However, NBI endoscopy is not commonly used in routine practice in Turkey. In addition, we did not assess the IM subtypes in accordance with recent studies, as it was not clinically significant.

In conclusion, patients with chronic gastritis with *H. pylori*, IM, and AG had an increased risk for gastric cancer. In these patients, screening endoscopy can detect early gastric cancer. Further prospective, long-term, large-scale follow up studies with immunohistochemical methods and double-blind assessment by two or three pathologists are necessary to assess gastric intestinal adenocarcinoma development in *H. pylori* patients.
